# Spread and dynamics of *Calicophoron daubneyi* and *Fasciola hepatica* in an extensively kept water buffalo herd and suitability of an ELISA for detecting antibodies against *F. hepatica*

**DOI:** 10.1007/s00436-026-08636-y

**Published:** 2026-01-30

**Authors:** Christoph Wenzel, Yury Zablotski, Gabriela Knubben-Schweizer, Christina Strube, Frank Weber

**Affiliations:** 1 Clinic for Ruminants with Ambulatory and Herd Health Services, Ludwig-Maximilians-Universität München, 85764 Oberschleissheim, Germany; 2 Institute for Parasitology, Centre for Infection Medicine, University of Veterinary Medicine Hannover, 30559 Hannover, Germany

**Keywords:** Liver fluke, Rumen fluke, Paramphistomes, Helminth infection, Biodiversity management, Wetland grazing

## Abstract

Water buffalo are ideal for grazing in restored wetlands due to their anatomical and behavioural adaptations. Unlike cattle or sheep, the water buffalo thrive in such habitats, feeding on rushes and reeds while helping maintain open landscapes. However, these moist environments also harbor risks, particularly parasitic infections by liver flukes (*Fasciola hepatica*) and the recently widespread rumen flukes, which require aquatic snails as intermediate hosts. For the present study faecal samples were taken once a year from 2016 to 2024 and monthly in two 12-month periods (2019/20 and 2022/23) from a water buffalo herd established in 2014 on a southern German farm and examined for fluke eggs with a sedimentation technique. In addition, climate data were obtained from a nearby weather station to analyse the impact of rainfall and sunshine hours on the fluke prevalence. To test whether blood samples can be used to detect infection with *F. hepatica*, residual quantities of blood samples from disease surveillance were tested for *F. hepatica* antibodies using a commercially available ELISA test. Copromicroscopical results showed that fluke prevalence rose significantly within a few years, with *F. hepatica* prevalence being always higher than *C. daubneyi* prevalence. Co-infections were common and seasonal patterns inconsistent. Fluke prevalence increased with rainfall and sunshine hours. In the herd, older water buffalo were more prone to liver flukes. The ELISA test for fascioliasis failed even though *F. hepatica* eggs were present in the faeces. The findings of the study highlight that extensively reared water buffalo are at high risk of trematode infection, which spreads rapidly throughout the herd and reaches high prevalence rates. The specific wallowing behaviour of water buffalo and favourable climatic conditions for parasite development may contribute to this development.

**Supplementary Information:**

The online version contains supplementary material available at 10.1007/s00436-026-08636-y.

## Introduction

In recent years, the rewetting of formerly drained natural areas such as fens, swamps and marshes has become increasingly important for nature conservation and climate protection reasons in Germany. Once these areas have been restored, they should be kept open by grazing with large herbivores in order to prevent trees and shrubs from regrowing and shading the smaller and rarer plant species of these wetlands. Studies show that grazing at such sites can be beneficial for conserving biodiversity (Enge 2009; Middleton et al. 2006; Wiegleb and Krawczynski 2010). Recently, water buffalo were used in these wet, boggy locations due to their wallowing behaviour (Wiegleb and Krawczynski 2010). This involves lying completely in a mud-hole with the head above the water to regulate the body temperature and acquire a generous coating of mud to prevent ectoparasites (Cockrill 1981). Their anatomical characteristics such as the shape of their claws and large, robust, and muscular physique makes them better adapted than domestic cattle and sheep to these areas. The latter species either avoid such locations (Middleton et al. 2006) or, if forced to graze there due to a limited food supply, could experience animal welfare issues. This is because the animals could become stuck in mud pools or starve while trying to survive on rush (Petermann et al. 2009). Water buffalo seem to be well-suited to grazing in wetlands from a biological viewpoint, due to their high level of adaptation to the environment, their high rate of food intake, and their low user costs (Sweers et al. 2013). From a health viewpoint, some diseases in water buffalo are said to occur less frequently (e.g. ixodid tick infestation) or be less harmful than in cattle (e.g. claw lesions and infections) (Cockrill 1981), although there is no reliable data on disease incidence. Basically they can be affected by the same diseases and parasites as other bovines from the Bovinae subfamily (Minervino et al. 2022). E.g. helminth infections occur in extensively reared water buffalo as well as in suckler cow herds (Gillandt et al. 2018; Rinaldi et al. 2009), and water buffalo can be infected by the same helminth species like gastrointestinal strongyles, *Strongyloides* spp., *Fasciola hepatica*, *Dicrocoelium dendriticum*, and Paramphistomidae, and especially trematodes can cause heavy infections due to the presence of the intermediate host snails in the areas where water buffalo wallow (Cockrill 1981). Therefore, in water buffalo the prevalence of trematodes (*F. hepatica*, rumen flukes) is higher than the prevalence of gastrointestinal nematodes (Kobak and Pilarczyk 2012; Liu et al. 2009). However, in extensive husbandry appropriate helminth control programmes are often neglected and therefore endoparasites cause economic losses as a consequence of deaths of infected animals (Petermann et al. 2009; Wiegleb and Krawczynski 2010), reduced rates of weight gains and the condemnation of infected organs after slaughter (Liu et al. 2009).

Rumen flukes have spread rapidly in Europe over the last two decades. *Calicophoron daubneyi* is the most abundant rumen fluke species and widespread in Germany (Forstmaier et al. 2021; Wiedermann et al. 2021), whereby the occurrence in grazing animals is particularly significant (Hecker et al. 2024). Rumen flukes have been mostly detected in cattle and sheep and in only a few cases in goats, new world camelids and water buffaloes. The latter has also been confirmed as final host for *C. daubneyi* (Ates and Umur 2021).

In Germany, the water buffalo (*Bubalus arnee* f. *bubalis*) has been bred for about 20 years (Spindler 2008; Sweers et al. 2013) and numbers of animals are rising (Minervino et al. 2020; Noce et al. 2021). A national official statistic about stocks and use of water buffalo is still lacking. It is assumed, that most of the water buffalo are kept for beef production, partly in combination with landscape conservation, and a smaller number for milk production, which requires intensive husbandry (Cringoli et al. 2009; Krawczynski et al. 2008).

Therefore, most of German water buffalo are probably kept in extensive systems, preferably under rough wet grassland conditions. Moisture on pastures is in the case of *F. hepatica* and *C. daubneyi* essential for the development of their intermediate host, the lymnaeid snail *Galba truncatula*. Although it has been shown for cattle and sheep that grazing on rewetted pastures did not increase endoparasite infection probability in the long term (May et al. 2022), it is to be expected that the water buffalo population is at a high risk of infection with trematodes. This paper aimed to gain insights into the distribution and dynamics of *C. daubneyi* and *F. hepatica* infections in an extensively kept water buffalo herd in southern Germany during a 9-year period. Moreover, data of rainfall and sunshine hours for the region were analysed on their association to fluke frequencies. Additionally, the suitability of an ELISA for the detection of antibodies against *F. hepatica* is reported.

## Materials and methods

### Study area and animals

**Fig. 1:**
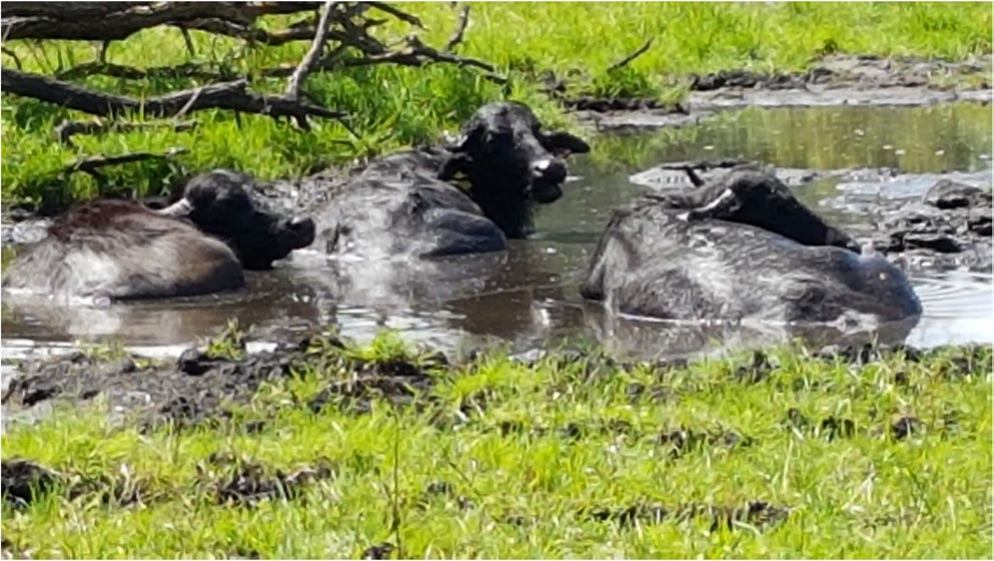
Water buffalo from the study wallowing in a mud hole

The study was conducted from 2016 to 2024 on a commercial suckler cow farm rearing water buffalo in the southern German region Swabia, located in the federal state of Bavaria. The herd was part of an annually routine parasitological monitoring conducted through the Clinic for Ruminants with Ambulatory and Herd Health Services, Ludwig-Maximilians-Universität (LMU) in Munich. The farm was located in a flat grassland area at an altitude of 440 m above sea level (ASL) on the eastern edge of the “Donauried” landscape. This is a valuable large-scale habitat containing alluvial forests, oxbow lakes, fens, grasslands, floodplains, ditches and litter meadows. The species-rich wet grasslands in this area were the focus of national nature conservation policy. Therefore, in 2014, the farmer started grazing six water buffalo on 50 hectares of damp, partially flooded grassland that had previously lain fallow as part of a nature conservation project. Ten years later, the herd had grown to 65 animals through purchase (11 breeding animals until 2018) and own offspring. Most calving took place in late winter and spring. The herd was pastured year-round and grazed rotationally on the whole area. Weaned calves were kept on separate pastures. The animals were treated once for liver flukes in 2018 to reduce egg output. There were no further indications for treatment with anthelmintics. Mineral supplement was offered throughout the year. In winter, hay was provided ad libitum as additional feed. A shelter provided protection against rain and wind. Only in very cold periods (< −10 °C) the animals were kept in a cowshed. Through their wallowing behaviour, the water buffalo created several water holes in the pastures in a very short time (Fig. 1). These holes provide water even in dry periods. The snail intermediate host of *F. hepatica* and *C. daubneyi*, *G. truncatula*, has been detected in several habitats on the pastures. The identification of the snail species was carried out based on shell morphology (Mehl 1932) directly on site.

### Faecal and blood sampling

In the frame of a national control programme for bovine alphaherpesvirus 1 (BoAHV-1), blood samples were taken from herd members older than 24 months once a year in summer (July or August). For this purpose, the animals were restrained in a mobile squeeze chute on the pasture and serum samples were collected either from the tail or the ear vein and subsequently sent to an official BoAHV-1 laboratory. Faecal samples were taken directly from the rectum at the same time. Collected samples were immediately stored in a cooler and transported on the same day to the laboratory of the Clinic for Ruminants with Ambulatory and Herd Health Services, LMU in Munich. Additionally, in the periods July 2019 to June 2020 and August 2022 to July 2023, faecal samples were taken monthly by the farmer from freshly excreted faeces, avoiding the collection of soil-touching portions. These samples were sent by mail to the laboratory within one day. After arrival in the laboratory, samples were stored at 4 °C and analysed within the next 14 days.

### Copromicroscopical examination and species identification

The faecal samples were examined for liver fluke eggs and rumen fluke eggs with sedimentation techniques in a semi-quantitative manner (0 = no eggs found, + = 1–10 eggs, ++ = 11–30 eggs, +++ > 30 eggs). From 2016 until June 2023, the classical sedimentation method was performed, for the analyses of the annual samples from 2023 to 2024 the Flukefinder^®^ technique (Flukefinder Diagnostic System, Soda Springs, ID, USA) was used. Briefly, the two sedimentation techniques were carried out as follows: For the classical technique approximately ten grams of faeces were weighed and thoroughly mixed with tap water in a mortar. The suspension was rinsed with 200 ml of tap water through a tea strainer into a 250 ml beaker (Simax^®^, Kavalierglass, Prague, Czech Republic) using a strong water jet. After 15 min of sedimentation, the supernatant was decanted, and the beaker refilled with tap water. This step was repeated three to four times until the supernatant appeared clear. For the Flukefinder^®^ technique two grams of faeces were mixed with 30 ml of tap water, poured into the Flukefinder^®^ and then rinsed with tap water three times. Debris retained on the wide-meshed sieve was discarded and the sediment captured on the narrow-meshed sieve was transferred into a 15 ml centrifuge tube. After sedimentation for two minutes, the supernatant was discarded and the centrifuge tube refilled with tap water. This step was performed three times. In both techniques the entire sediment was supplemented with one to two drops of 1% methylene blue (Merck, Darmstadt, Germany) and examined in a Petri dish under a microscope (Leica DM IL, Wetzlar, Germany) at 40x magnification. To this end, the Petri dish was examined thoroughly and a maximum of 31 eggs were counted in order to assign the infection to a corresponding category (0, +, ++, +++). Rumen fluke eggs and liver fluke eggs were differentiated by their colour: greyish-pale = rumen fluke eggs vs. yellow-brownish = fasciolid eggs. In 2019, rumen fluke eggs were isolated from faecal samples for subsequent molecular species identification by targeting the internal transcribed spacer ITS-2 region as described previously (May et al. 2019). The sequenced ITS-2 PCR product showed 100% identity with *C. daubneyi* (GenBank accession number KP201674).

### Detection of *Fasciola hepatica* antibodies using a commercially available ELISA

In 2019, serum residues from BoAHV-1 tests of 12 randomly selected water buffalo were examined for anti-*F. hepatica* antibodies using the commercially available IDEXX Fasciolosis Verification Test (IDEXX, Kornwestheim, Germany). The samples were analysed according to the manufacturer’s instructions, i.e. testing the coated well with the provided antigen (f2-antigen, a fraction purified from E/S-antigen of *F. hepatica*) against a second uncoated well. The resulting absorbance from the uncoated well was subtracted from the measured absorbance of the f2-antigen-coated well. Based on the ratio of the absorbance value of the positive samples (S) to the mean absorbance value of the positive control (P), results were expressed as sample to positive percentage (S/P%) for each sample.

### Data sources

Climate data (i.e. rainfall, sunshine hours in May, June and July [MJJ]) were obtained from the next weather station of the German Meteorological Service (Deutscher Wetterdienst), which was 40 km away from the pastures. Rainfall and sunshine hours (MJJ) were selected as important climatic factors influencing the activity of *G*. *truncatula* (Jones et al. 2017). The age of the animals was taken from the cattle passport.

### Statistical analysis

The data was prepared using Microsoft Excel 2016 (Microsoft Corporation, Redmond, WA, USA). Prevalence and egg excretion of *F. hepatica* and *C. daubneyi* were reported descriptively. Statistical analyses of associations between prevalence, age and climatic data were performed using R (The R Foundation for Statistical Computing, Vienna, Austria). Because of repeated measures, mixed-effects logistic regression was initially considered; however, the mixed-effects model failed to converge due to the low number of observations within the groups and categories of interest. Therefore, we used binomial Bayesian logistic regression without random effects. This approach leverages the robustness of Bayesian estimation for low-probability outcomes while providing frequentist-style 95% confidence intervals and *P*-values to verify if age, rainfall, and sunshine hours (MJJ) influence fluke egg prevalence for each parasite (*Fasciola hepatica* and *Calicophoron daubneyi*) separately (function “bayesglm” in the “arm” package in R) (Gelman and Su 2024). This approach, incorporating weakly informative priors, was selected to stabilise coefficient estimates and improve inference reliability, as standard logistic regression could produce unstable estimates and excessively wide confidence intervals due to low event counts. Significance was set at *P* ≤ 0.05.

## Results

### Distribution and dynamics of *Calicophoron daubneyi* and *Fasciola hepatica*

**Fig. 2:**
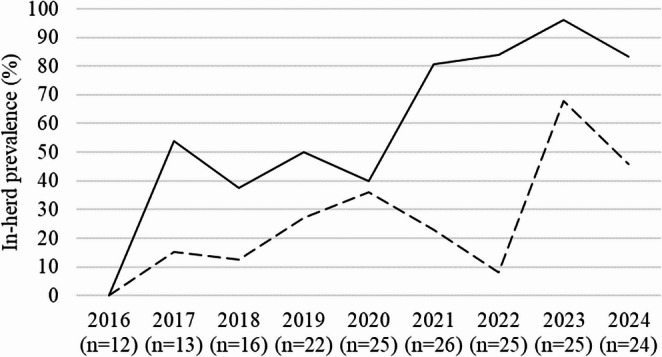
In-herd prevalences of *Fasciola hepatica* (solid line) and *Calicophoron daubneyi* (dashed line) in a water buffalo herd over a nine-year period (2016–2024)

The number of faecal samples from water buffalo older than 24 months rose from 12 in 2016 to 13 (2017), 16 (2018), 22 (2019), 25 (2020) to 26 samples in 2021, and then decreased from 25 (2022 and 2023) to 24 samples in 2024. The sample size varied from 2018 onwards, as it was not possible to sample all adults every year. In 2016 no trematode eggs were found in the faecal samples. Then the in-herd prevalence for *F. hepatica* increased with two small drops in 2018 and 2020 to a peak of 96% in 2023. *Calicophoron daubneyi* eggs were first discovered in 2017 and in-herd prevalence rose to 36% in 2020, declined then to 8% in 2022 and increased again to 68% in 2023 (Fig. 2). On average over the annual surveys from 2016 to 2024, the number of observations in the egg excretion categories ‘0’ or ‘+’ were higher than in the categories ‘++’ or ‘+++’ for both flukes (Table 1).

**Table 1:** Raw counts (with percentages in brackets) of the different egg excretion quantities (0, +, ++, +++) for *Fasciola* (*F*.) *hepatica* and *Calicophoron* (*C*.) *daubneyi* from all annual copromicroscopical examinations (*n* = 188) from 2016 to 2024 in a water buffalo herd. The number of samples varied between years (Min = 12, Max = 26)

	Egg excretion score			
	0	+ (< 10 eggs)	++ (11–30 eggs)	+++ (> 30 eggs)
*F. hepatica*	68 (34%)	82 (67%)	37 (74%)	1 (50%)
*C. daubneyi*	133 (66%)	41 (33%)	13 (26%)	1 (50%)

### Co- and new infections

Co-infections showed an undulating course and peaked in 2023 with 68% (Table 2). The average co-infection rate in the study period was 23.6%. New infections increased to 100% at the second copromicroscopical examination. Thereafter frequency of new infections with *F. hepatica* decreased, while those of *C. daubenyi* increased again to 83%. In spring 2018 three cows co-infected with *F. hepatica* (++) and rumen flukes (+++), which were selected by the farmer, received an oral drench with oxyclozanide (10 mg/kg BW), and two cows mono-infected with *F. hepatica* (+) received an oral drench with albendazole (10 mg/kg BW), each at the dosage recommended by the manufacturer. Faecal samples of four cows were negative for *F. hepatica* in the subsequent investigation (July 2018), but were positive again in 2019. No new infections were detected during the last examination in 2024 (Table 2). All animals examined between 2016 and 2024 tested positive for both types of fluke eggs during either the monthly or annual examination, with the exception of one cow, in which only *F. hepatica* eggs were detected. More animals were positive for *F. hepatica* eggs (*n* = 19) than for *C. daubneyi* eggs (*n* = 3) on at least three consecutive examinations (Suppl. Table 1).

**Table 2:** Frequencies of co- and new infections with *Fasciola hepatica* and *Calicophoron daubneyi* in a water buffalo herd from 2016 to 2024

Year	Co-Infection (%)	New Infection (%)	
		*Fasciola hepatica*	*Calicophoron daubneyi*
2016	0	0	0
2017	15.4	100.0	100.0
2018	6.3	50.0	50.0
2019	22.7	54.5	83.3
2020	8.0	40.0	44.4
2021	23.1	23.8	16.7
2022	8.0	4.8	0
2023	68.0	4.2	35.3
2024	37.5	0	0

### Seasonality

**Fig. 3:**
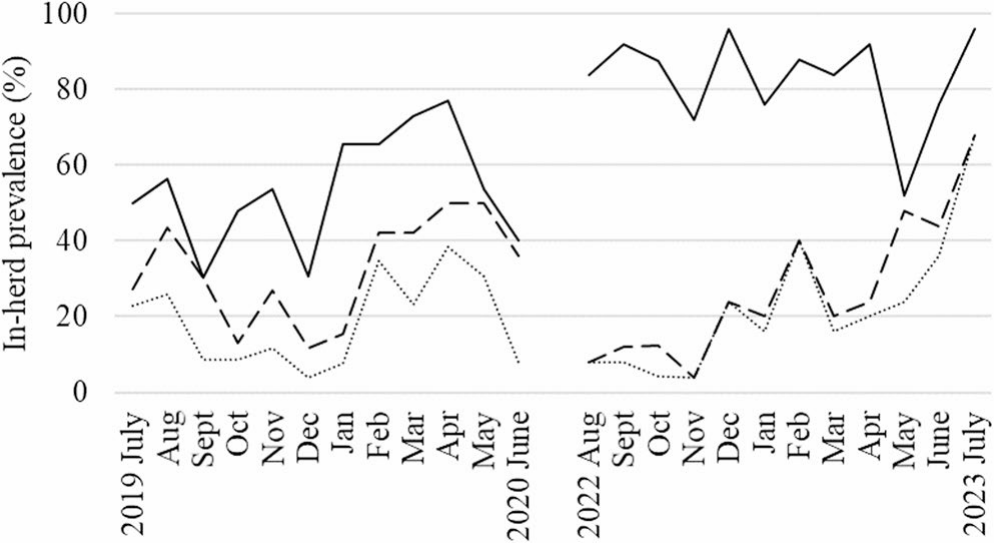
Monthly distribution of *Fasciola hepatica* (solid line), *Calicophoron daubneyi* (dashed line), and co-infection frequency (dotted line) in a water buffalo herd over two one-year periods (2019/20 and 2022/23). Co-infected samples are also included in the percentages of each single parasite

At any given time of the 12-month-periods in 2019/20 and 2022/23, *F. hepatica* infection was more frequent than *C. daubneyi* infection in the water buffalo herd, except for September 2019, where frequencies were equal. Otherwise, the infection rates went up and down, but followed a similar course except for October 2019 and May 2023 where the course of both fluke frequencies was contrary. In 2019/20 both fluke species peaked from January to May, but not in the period 2022/23, where the *F. hepatica* frequency peaked in several months throughout the period, and the *C. daubneyi* frequency in February, May and July 2023. The average frequency of rumen flukes was similar in 2019/20 (32.4%) and 2022/23 (27.0%). In contrast, liver fluke frequency differed clearly between both periods (2019/20: 53.7% vs. 2022/23: 83.0%). Overall, the average frequency of co-infection with both parasites was similar in the two periods (2019/20: 18.7% vs. 2022/23: 22.3%) (Fig. 3). In the monthly examinations the number of samples varied between 22 and 29, as it was not possible to sample all animals each time. From July 2019 to June 2020 and from August 2022 to July 2023 no or low egg excretion (‘0’ or ‘+’) was more frequently observed than score ‘++’ or ‘+++’ for both parasites but the highest egg excretions were seen in the latter period for liver fluke eggs (Table 3).

**Table 3:** Raw counts (with percentages in brackets) of different egg excretion quantities (0, +, ++, +++) for *Fasciola* (*F.*) *hepatica* and *Calicophoron* (*C.*) *daubneyi* from all monthly copromicroscopical examinations of the periods July 2019 to June 2020 (*n* = 309) and August 2022 to July 2023 (*n* = 299) in a water buffalo herd. The number of samples varied in some months (2019/20: Min = 22, Max = 29, 2022/23: Min = 24, Max = 25)

		Egg excretion score			
Period	Parasite	0	+ (< 10 eggs)	++ (11–30 eggs)	+++ (> 30 eggs)
2019 -	*F. hepatica*	151 (41%)	150 (63%)	7 (58%)	1 (100%)
2020	*C. daubneyi*	216 (59%)	88 (37%)	5 (42%)	0
2022 -	*F. hepatica*	51 (19%)	186 (75%)	35 (78%)	27 (65%)
2023	*C. daubneyi*	217 (81%)	63 (25%)	10 (22%)	9 (35%)

### Association of fluke egg odds ratios (OR) with sunshine hours (MJJ), total rainfall and age

**Fig. 4:**
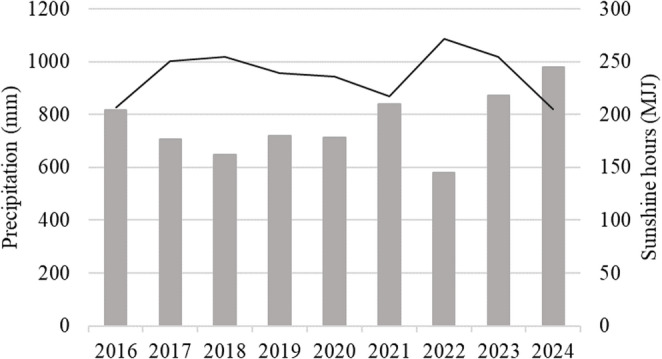
Annual precipitation in mm (light grey columns) and sunshine hours (dark grey line) in the months of May, June, July (MJJ). Data obtained from the nearest weather station to the pasture of a water buffalo herd in southern Germany

The climate in the region was temperate and annual average values in the study period were 9.8 °C temperature (minimum − 14.2 °C – maximum 33.5 °C), 79.2% humidity (76.0% – 81.9%), and 763.8 mm rainfall (580.2–978.1 mm). The sunshine hours in the months of May, June and July (MJJ) averaged 237 h per year (minimum 205 h – maximum 272 h) (Fig. 4).

The multivariable model showed that the liver fluke frequency increased significantly with age, but the rumen fluke frequency did not. Both frequencies increased if sunshine hours (MJJ) and total rainfall increased (Table 4).

**Table 4:** Multivariable Bayesian logistic regression models showing the association of fluke frequencies with sunshine hours in May, June and July (MJJ), total rainfall per year and age of animals in a water buffalo herd

	*Fasciola hepatica*			*Calicophoron daubneyi*		
Characteristic	OR	95%-CI	*P*-value	OR	95%-CI	*P*-value
Sunshine hours (MJJ)	1.05	1.01, 1.09	0.007	1.04	1.01, 1.06	0.003
Total rainfall per year (mm)	1.01	1.00, 1.01	0.030	1.01	1.01, 1.01	< 0.001
Age in years	1.58	1.31, 1.89	< 0.001	0.96	0.83, 1.12	0.634

Abbreviations: *OR* Odds Ratio, *CI* Confidence Interval

### Detection of *Fasciola hepatica* antibodies using a commercially available ELISA

The serological test for *F. hepatica* antibodies was negative in all cases, although liver fluke eggs were detected in the faeces at the same time or before (Table 5).

**Table 5:** Serological results of a *F. hepatica*-ELISA compared to copromicroscopical results from the same test day and from the prior sampling in a water buffalo herd

Animal No.	Copromicroscopical result 2018		Copromicroscopical result 2019		ELISA result 2019 (S/P%)
	Rumen fluke	*Fasciola hepatica*	Rumen fluke	*Fasciola hepatica*	
1	0	0	0	+	1.2
2	0	+	0	0	2.3
3	0	+	0	0	0.1
6	0	+	0	0	0.6
8	0	0	+	+	0.4
9	0	0	0	+	0.0
11	0	0	0	0	0.5
13	-	-	+	+	0.2
17	0	0	+	0	0.1
19	-	-	0	0	1.8
20	-	-	0	0	0.2
23	-	-	+	+	0.2

S/P = Sample-to-Positive Ratio, Interpretation of the ELISA results: S/P% ≤ 30% = negative

0 = no eggs found, + = fluke eggs found, - = no sample available

## Discussion

Water buffalo husbandry is a niche in temperate climates of Germany, but seems to be a successful way to fulfil recommendations concerning nature conservation, i.e. keeping wet grassland areas open (Wiegleb and Krawczynski 2010). At the same time, the wetlands harbour the risk of infection with rumen flukes and liver flukes, as their life cycle is linked to aquatic intermediate host snails. The most abundant rumen fluke in Germany, *C. daubneyi*, has the same intermediate host, the lymnaeid snail *G. truncatula* as the liver fluke *F. hepatica* (Jones et al. 2015).

There is no information available on the infection rates of rumen and liver flukes among the water buffalo population in Germany, but our study showed, how a naïve herd can be infected in a short time and reach prevalences similar to herds in subtropic and tropic climates, where water buffalo are crucial for agricultural work (Liu et al. 2009; Tookhy et al. 2024). On the second annual routine parasitological monitoring and three years after the introduction into the grassland area, infected animals were firstly detected in the studied herd. As no ruminants previously grazed on the pastures, the flukes were probably introduced by purchased animals. The reason for the quick rise in in-herd prevalences was the presence of *G. truncatula* in several habitats of the pastures, where water buffalo were grazing and wallowing. In the observation period from 2016 to 2024, the annual *F. hepatica* in-herd prevalence fluctuated between 37.5% and 96.0%, and those of *C. daubneyi* between 8.0% and 68.0%. The variation in in-herd prevalence rates is affected by various factors including climate, herd size, and the use of anthelmintics against *F. hepatica* (Jones et al. 2017). After the anthelmintic treatment in 2018 not only the liver fluke in-herd prevalence declined but also the rumen fluke in-herd prevalence, although neither the dosage of oxyclozanide used (10 mg/kg) nor the active ingredient albendazole is appropriate for rumen flukes (Arias et al. 2013). As the farmer steadily increased the size of his herd through purchases and home-bred animals, the proportion of infected animals also rose. Furthermore, it cannot be ruled out that the different sedimentation methods have influenced the egg counts, as the Flukefinder^®^ technique determines higher egg counts than the classical sedimentation technique (Hecker et al. 2024). In 2022, the prevalence of *C. daubneyi* reached its lowest level in the observation period for no apparent reason. Although the risk of infection with both trematodes increased due to the wallowing behaviour of the water buffalo, eggs were predominantly found in the lower categories (+, ++) than in the high category (+++). For German dairy cows low faecal egg counts were also shown (May et al. 2019), but a direct comparison between the studies was not possible due to the different egg count methods. Therefore, quantitative egg counts from water buffalo would be needed to evaluate the egg output for this ruminant species.

The observed prevalence of *F. hepatica* was always higher in the herd than the prevalence of *C. daubneyi*. This was not unexpected, as it reflects the situation in southern German cattle and sheep herds, where the herd prevalence of *F. hepatica* was higher than that of *C. daubneyi* (Alstedt et al. 2022; Forstmaier et al. 2021). In extensive herds in Poland and China, the same distribution of fluke prevalences were found (Kobak and Pilarczyk 2012; Liu et al. 2009). In contrast, intensive buffalo breeding for dairy purposes together with the regular use of anthelminthics seemed to be responsible for low prevalences of *F. hepatica* and *C. daubneyi* (ea. 7.1%) (Rinaldi et al. 2009). Since there was no helminth control programme in our water buffalo herd, this probably contributed to the high prevalence of *F. hepatica*.

Co-infections of liver and rumen flukes were observed at all positive sampling events. Frequencies reached mostly higher percentages than in Malaysian water buffalo (13.7%) and Irish cattle and sheep (ea. ≤ 10%), where rumen flukes are more prevalent than *F. hepatica* (Naranjo-Lucena et al. 2018; Tookhy et al. 2024). The curve of co-infection shown in Fig. 3 largely followed the course of the *F. hepatica* curve and the *C. daubneyi* curve in 2019/20, but in 2022/23 it followed only the rumen fluke curve. In a study with a large data set from veterinary surveillance the co-infection curve of cattle mirrors that of the liver fluke, but in sheep that of both flukes (Naranjo-Lucena et al. 2018). Data from Irish sheep strongly suggest that co-infection led to higher liver fluke egg counts (Munita et al. 2019b). In contrast, in German dairy cows co-occurrence of both fluke species does not appear to affect egg output (Hecker et al. 2024). Unfortunately, our data set is too small to contribute substantially to the literature on the relationship between *F. hepatica* and *C. daubneyi* in co-infected water buffalo.

In 2019/20 both fluke frequencies peaked several times with prevalences of *F. hepatica* above 60% from January to April. In the period 2022/23 the liver fluke prevalence was throughout above 70% from August to April. A clear seasonal pattern as in the case of Irish sheep with consistent peaks during the winter season for both fluke species could not be observed. In the same study, rumen fluke infection rates of cattle peaked also during the winter season, but patent liver fluke infections showed no distinct seasonality (Naranjo-Lucena et al. 2018). Although the former results were based on diagnostic submissions, which was a potential bias, it remains unclear, if water buffalo have different seasonal patterns compared to other host species. Therefore, to better assess the seasonality of trematodes in water buffalo, one sample occasion per season over several years would be necessary.

Logistic regression showed that frequencies of both fluke species increased when sunshine hours (MJJ) and rainfall increased. Both climatic factors are important for the development of both flukes in the environment (Vignoles et al. 2014), but in a large study from Wales no association between *F. hepatica* prevalence in cattle and sunshine hours (MJJ) has been shown (Jones et al. 2017). *Calicophoron daubneyi* eggs are known to be more dependent on light as a hatching stimulus compared with *F. hepatica* eggs (Chryssafidis et al. 2015), which could result in higher hatching success rates. A climate-driven disease model that takes precipitation and temperature into account predicted that the risk of fasciolosis has increased significantly in southern Germany (Caminade et al. 2015), but due to the prevalence found in our herd it seemed questionable whether this would have a similar effect on *C. daubneyi*.

Although both trematodes share the same intermediate host (*G. truncatula*) and thus have a similar epidemiology, the reason for the differing in-herd prevalence of the flukes may also lie within the host itself. The prevalence of fasciolosis, but not of paramphistomidosis, increased significantly with the age of the animals, which agrees with a report from Spain (Arias et al. 2011). But in southern German dual-purpose dairy cows older than six years the prevalence of both trematodes were higher than in younger cows (Teschner et al. 2025). Although the results were to be expected in our herd, it remains unclear whether our findings were due to different exposure to infective fluke stages or to an immune response by the host. In the present study more water buffalo were found with consistent *F. hepatica* egg excretion than with *C. daubneyi* egg excretion. This was surprising, as chronic hepatic fascioliosis should lead to cholestasis (Tharwat 2012), which impedes bile flow. However, the significantly more frequent continuous excretion may have led to greater contamination of habitats with *F. hepatica* eggs than with *C. daubneyi* eggs.

Liver fluke specific ELISAs have been developed and are being routinely used in cattle (Munita et al. 2019a). The IDEXX Fasciolosis Verification test uses the *F. hepatica* specific f2 antigen and has shown to be very reliable with reported sensitivity and specificity ratios of 100% in experimental infected young cattle (Kuerpick et al. 2013). As the test was validated for bovine and ovine sera and on bovine milk’ (Manual of IDEXX Fasciolosis Verification) we used the test with water buffalo serum since the term ‘bovine’ does not allow for a clear zoological classification within the subfamily Bovinae (Sambraus 2006). Moreover, for binding the f2 antibody a so-called ‘anti-ruminant antibody enzyme conjugate’ was used (Manual of IDEXX Fasciolosis Verification), which should be specific to ruminants and also include the water buffalo. It was therefore expected that this conjugate would bind the f2 antibodies circulating in the blood of infected water buffalo to antigen-antibody immune complexes and thus detecting an infection with *F. hepatica*. However, our study showed that the ELISA cannot be used with water buffalo serum without further modification. It is likely that the detection antibody anti-ruminant immunoglobulin G (IgG) used in the test system needs to be replaced by an anti-Bubalus IgG in order to use the test on water buffalo.

## Conclusion

It can be concluded that the extensively reared water buffalo herd was at high risk of becoming infected with trematodes because of their behaviour of wallowing in areas where *G. truncatula* was common and the optimised development of trematodes in favourable climatic conditions. In this case, fencing off habitats is not an option, as this would hinder the water buffalo’s wallow behaviour. Although the egg excretion rates in our water buffalo herd were very high in some cases, the farmer did not report any clinical symptoms (e.g., diarrhoea, anorexia or emaciation) and was not concerned about the infections. In herds free of trematodes, purchased animals should be examined for trematode eggs before they are released to pasture with potential intermediate host habitats in order to prevent the introduction of flukes into the herd.

## Supplementary Information

Below is the link to the electronic supplementary material.

Supplementary file1 (DOCX 34.5 KB)

## Data Availability

All data supporting the findings of this study are available within the paper and its Supplementary Information.
