# Supplementary material for: Spread and dynamics of Calicophoron daubneyi and Fasciola hepatica in an extensively kept water buffalo herd and suitability of an ELISA for detecting antibodies against F. hepatica
Source: Parasitol Res. 2026 Jan 30;125(1):13. doi: 10.1007/s00436-026-08636-y (PMC12868077; doi:10.1007/s00436-026-08636-y)
Supplement: Supplementary file 1 — Supplementary file1 (DOCX 34.5 KB) [file 436_2026_8636_MOESM1_ESM.docx]

Suppl. Table 1: Positivity (+) for *Fasciola* *hepatica* and *Calicophoron daubneyi* in copromicroscopical examination of samples from water buffalo between 2016 and 2024.

| Animal No. | Fluke species | 2016 | 2017 | 2018 | 2019 | 2020 | 2021 | 2022 | 2023 | 2024 |
| --- | --- | --- | --- | --- | --- | --- | --- | --- | --- | --- |
| 1 | *F. hepatica* | 0 | + | 0 | + | 0 | + | + | + | + |
|  | *C. daubneyi* | 0 | 0 | 0 | 0 | + | 0 | + | + | + |
| 2 | *F. hepatica* | 0 | 0 | + | 0 | 0 | 0 | + | + | + |
|  | *C. daubneyi* | 0 | 0 | 0 | 0 | + | 0 | 0 | + | 0 |
| 3 | *F. hepatica* | 0 | + | + | 0 | 0 | + | + | + | + |
|  | *C. daubneyi* | 0 | + | 0 | 0 | 0 | 0 | + | + | 0 |
| 4 | *F. hepatica* | 0 | 0 | + | 0 | 0 | + | 0 | + | + |
|  | *C. daubneyi* | 0 | 0 | 0 | 0 | 0 | 0 | 0 | 0 | 0 |
| 5 | *F. hepatica* | 0 | + | + | + | + | + | + | + | + |
|  | *C. daubneyi* | 0 | 0 | + | 0 | 0 | 0 | 0 | 0 | 0 |
| 6 | *F. hepatica* | 0 | + | + | 0 | + | + | + | + | + |
|  | *C. daubneyi* | 0 | 0 | 0 | 0 | 0 | 0 | 0 | 0 | 0 |
| 7 | *F. hepatica* | 0 | 0 | + | + | + | + | + | + | + |
|  | *C. daubneyi* | 0 | 0 | 0 | 0 | 0 | 0 | 0 | + | 0 |
| 8 | *F. hepatica* | 0 | + | 0 | + | + | + | + | + | + |
|  | *C. daubneyi* | 0 | 0 | 0 | + | 0 | 0 | 0 | 0 | + |
| 9 | *F. hepatica* | 0 | 0 | 0 | + | 0 | + | 0 | + | + |
|  | *C. daubneyi* | 0 | 0 | 0 | 0 | 0 | 0 | 0 | + | 0 |
| 10 | *F. hepatica* | 0 | + | 0 | + | 0 | + | + | + | + |
|  | *C. daubneyi* | 0 | + | 0 | 0 | 0 | 0 | 0 | + | 0 |
| 11 | *F. hepatica* | 0 | 0 | 0 | 0 | 0 | + | + |  |  |
|  | *C. daubneyi* | 0 | 0 | 0 | 0 | 0 | 0 | 0 |  |  |
| 12 | *F. hepatica* | 0 | + | x | x | + | + | x | + | + |
|  | *C. daubneyi* | 0 | 0 | x | x | 0 | 0 | x | 0 | 0 |
| 13 | *F. hepatica* |  | 0 | x | + | + | + | 0 | + | + |
|  | *C. daubneyi* |  | 0 | x | + | + | + | 0 | + | + |
| 14 | *F. hepatica* |  |  | 0 | + | 0 | 0 | + | + | 0 |
|  | *C. daubneyi* |  |  | 0 | 0 | 0 | 0 | 0 | + | + |
| 15 | *F. hepatica* |  |  | 0 | + | 0 | 0 | + | 0 | + |
|  | *C. daubneyi* |  |  | 0 | + | + | 0 | 0 | 0 | + |
| 16 | *F. hepatica* |  |  | 0 | 0 | 0 | 0 | 0 | + | 0 |
|  | *C. daubneyi* |  |  | 0 | 0 | 0 | 0 | 0 | + | 0 |
| 17 | *F. hepatica* |  |  | 0 | 0 | 0 | + | + | + | + |
|  | *C. daubneyi* |  |  | 0 | + | + | + | 0 | + | + |
| 18 | *F. hepatica* |  |  | 0 | + | + | + | + | + |  |
|  | *C. daubneyi* |  |  | + | + | 0 | + | 0 | + |  |
| 19 | *F. hepatica* |  |  |  | 0 | 0 | + | + | + | 0 |
|  | *C. daubneyi* |  |  |  | 0 | + | + | 0 | + | + |
| 20 | *F. hepatica* |  |  |  | 0 | + | + | + | + | + |
|  | *C. daubneyi* |  |  |  | 0 | + | 0 | 0 | + | 0 |
| 21 | *F. hepatica* |  |  |  | 0 | x | + | + | + | + |
|  | *C. daubneyi* |  |  |  | 0 | x | 0 | 0 | 0 | 0 |
| 22 | *F. hepatica* |  |  |  | 0 | + | + | + | + | + |
|  | *C. daubneyi* |  |  |  | 0 | 0 | + | 0 | 0 | + |
| 23 | *F. hepatica* |  |  |  | + | 0 | + | + | + | + |
|  | *C. daubneyi* |  |  |  | + | + | 0 | 0 | + | + |
| 24 | *F. hepatica* |  |  |  |  | 0 | + | + | + | + |
|  | *C. daubneyi* |  |  |  |  | + | + | 0 | + | + |
| 25 | *F. hepatica* |  |  |  |  | + | + | + | + | 0 |
|  | *C. daubneyi* |  |  |  |  | 0 | 0 | 0 | + | 0 |
| 26 | *F. hepatica* |  |  |  |  | 0 | 0 | + | + | + |
|  | *C. daubneyi* |  |  |  |  | 0 | 0 | 0 | + | + |

0 = no eggs found, + = eggs found, x = sample missing, blank = animal not in herd
